# Biomolecules and Cardiovascular Diseases in Women

**DOI:** 10.3390/biom12121750

**Published:** 2022-11-24

**Authors:** Barbara Ghinassi, Angela Di Baldassarre, Clara Crescioli

**Affiliations:** 1 Reprogramming and Cell Differentiation Lab, Center for Advanced Studies and Technology (CAST), 66100 Chieti, Italy; 2 Department of Medicine and Aging Sciences, University “G. d’Annunzio” of Chieti-Pescara, 66100 Chieti, Italy; 3 Department of Movement, Human and Health Sciences, University of Rome “Foro Italico”, 00135 Rome, Italy

Although cardiovascular diseases (CVD) are the leading cause of non-communicable diseases-dependent death worldwide, their effects are still largely underestimated in women [1]. The existing differences between men and women have made CVD risk management more challenging, and this is pushing scientists to underline sex differences in the mechanisms of CVD progression and to explore new sex-specific therapeutic strategies. Age-associated modifications in body composition, hormonal and metabolic factors, as well as a decline in physical activity are all involved in the increased risk of CVD development [2,3]. Women experience a dramatic rise in cardiovascular (CV) events after menopause. The estrogen reduction during the menopausal transition is indicated as the major trigger for the increased risk of CVD, but growing evidence in literature highlights that, probably, the hormonal change per se is not enough to drive alone CVD development and progression in women. This Special Issue highlights the emerging role of some biomolecules highly impacting CV system in women. Crescioli [4] clearly reviewed the protective role of vitamin D in female cardiac health, discussing that vitamin D controls together with estrogens some intracellular signaling paths [5], so that the unfavorable association of hypoestrogenism-D hypovitaminosis likely converges towards maladaptive cardiomyocyte remodeling and contributes to increase CVD risk in post-menopause. Da Venezia et al. [6] assessed the association between periodontal diseases and CV risk based on serum high-sensitivity C-reactive protein, plainly showing a link between periodontitis, that might act as a non-traditional CV risk factor, and a low-grade systemic inflammation mediated by C-reactive protein. They showed the systemic and periodontal correlation in women’s gingival crevicular fluid and its impact on CV risk. In the past decades, attention has been focused on several pollutants that potentially disrupt human health by interfering with hormonal pathways, specifically impacting several non-communicable diseases, including CVD. Migliaccio et al. [7] deeply reviewed the potential alteration induced by these chemicals, classified as endocrine disruptors, in the attempt to characterize a potential role in the cellular and molecular mechanisms involved in the atheromatous degeneration process and CVD progression. Megiorni et al. [8] highlighted the importance of sex/gender medicine in tailoring personalized programs for COVID-19 prevention, clinical evaluation and treatment. They discussed the potential mechanisms accounting for sex/gender influence in vulnerability to COVID-19 infection and related CV manifestations, focusing on sex-dependent variability in cardiac- thrombotic- and inflammatory biomarkers in the evaluation of COVID-19 infection and prognosis. Moreover, Pandurevic et al. [9] presented the available evidence on the potential implications of some biomediators, in particular related to hyperandrogenism, estrogen-progesterone imbalance, insulin resistance, and low steroid hormone-binding globulin, in the processes leading to CV disease in polycystic ovary syndrome, with the final aim to propose a more accurate CV risk assessment. Bengiano and colleagues [10] highlighted how a history of hypertensive disorders in pregnancy is linked with an increased risk of CVD, suggesting that vascular pathology during gestation and CVD may share a common etiology/risk factors or that the future occurrence of a CVD may be the consequence of endothelial dysfunction generated by pregnancy-induced hypertension that persists after delivery. Moreover, Kryczka et al. [11] extensively reviewed how the synergistic interplay between thrombosis, inflammation, and the renin–angiotensin system seems to influence coronary artery disease and atherosclerotic plaque formation more significantly in young women than in men, suggesting that the diverse pathological pathways contributing to premature coronary artery disease differ in women and men. Yang and colleagues [12] investigated the link between mental health, brain activation, hypothalamic–pituitary–adrenal axis, autonomic nervous system, blood pressure and immune system associated with CV health in women and discussed the effects of mind–body intervention in modulating these factors. Trtica Majnarić et al. [13] analyzed in the lifetime course, with a particular regard to the menopause, sex specific differences of the neutrophil-to-lymphocyte ratio within immune and inflammatory mechanisms. High-sensitivity cardiac troponin assays have become gold standard for diagnosing acute and chronic myocardial injury; however, clinical investigations still debate on the use of different cut-offs for men and women [14,15]. Bisaccia and colleagues [16] deeply reviewed the pivotal role of cardiac troponin as a prognostic marker of disease. Sex-based differences in cardiac troponin levels are further explored, with regard to the prognostic significance of sex-specific diagnostic thresholds of high-sensitivity cardiac troponin assays versus common cut-offs in men and women undergoing CVD risk assessment, and to the clinical utility of high-sensitivity cardiac troponin assays for CV disease prevention in women. 

Since, for many decades, CVD research has been performed primarily on men, this Special Issue aims to encourage a translational approach involving contributions from basic and clinic researchers that would lead to a sex/gender-tailored medicine with the identification of sex specific biomolecules profiling, with clinical relevance for predictive, diagnostic or prognostic purposes for CVD prevention in both sexes.
